# Clinical considerations and key issues in the management of patients with Erdheim-Chester Disease: a seven case series

**DOI:** 10.1186/s12916-014-0221-3

**Published:** 2014-12-01

**Authors:** Roei D Mazor, Mirra Manevich-Mazor, Anat Kesler, Orna Aizenstein, Iris Eshed, Ronald Jaffe, Yakov Pessach, Ilan Goldberg, Eli Sprecher, Iris Yaish, Alexander Gural, Chezi Ganzel, Yehuda Shoenfeld

**Affiliations:** The Zabludowicz Center for Autoimmune Diseases, Sheba Medical Center, Tel Hashomer, Israel; Sackler Faculty of Medicine, Tel Aviv University, Tel Aviv, Israel; Department of Ophthalmology, Neuro-ophthalmology Unit, Tel Aviv-Sourasky Medical Center, Tel Aviv, Israel; Department of Radiology, Neuroradiology Unit, Tel Aviv-Sourasky Medical Center, Tel Aviv, Israel; Department of Diagnostic Imaging, Sheba Medical Center, Tel Aviv University, Tel Hashomer, Israel; Department of Pathology, University of Pittsburgh, School of Medicine and Children’s Hospital, Pittsburgh, USA; Department of Dermatology, Tel-Aviv Sourasky Medical Center, Tel-Aviv, Israel; Institute of Endocrinology, Metabolism and Hypertension, Tel Aviv-Sourasky Medical Center, Tel Aviv, Israel; Department of Hematology, Hadassah University Hospital, Jerusalem, Israel; Department of Hematology, Shaare Zedek Medical Center, Jerusalem, Israel

**Keywords:** Erdheim Chester, *BRAF*, *NRAS*, Vemurafenib, Interferon-α, Histiocytosis

## Abstract

**Background:**

Erdheim-Chester Disease (ECD), a non Langerhans’ cell histiocytosis of orphan nature and propensity for multi-systemic presentations, comprises an intricate medical challenge in terms of diagnosis, treatment and complication management.

**Objectives:**

The objectives are to report the clinical, radiological and pathological characteristics, as well as cardinal therapeutic approaches to ECD patients and to provide clinical analyses of the medical chronicles of these complex patients.

**Methods:**

Patients with biopsy proven ECD were audited by a multi-disciplinary team of specialists who formed a coherent timeline of all the substantial clinical events in the evolution of their patients’ illness.

**Results:**

Seven patients (five men, two women) were recruited to the study. The median age at presentation was 53 years (range: 39 to 62 years). The median follow-up time was 36 months (range: 1 to 72 months). Notable ECD involvement sites included the skeleton (seven), pituitary gland (seven), retroperitoneum (five), central nervous system (four), skin (four), lungs and pleura (four), orbits (three), heart and great vessels (three) and retinae (one). Prominent signs and symptoms were fever (seven), polyuria and polydipsia (six), ataxia and dysarthria (four), bone pain (four), exophthalmos (three), renovascular hypertension (one) and dyspnea (one). The *V600E BRAF* mutation was verified in three of six patients tested. Interferon-α treatment was beneficial in three of six patients treated. Vemurafenib yielded dramatic neurological improvement in a *BRAF* mutated patient. Infliximab facilitated pericardial effusion volume reduction. Cladribine improved cerebral blood flow originally compromised by perivenous lesions.

**Conclusions:**

ECD is a complex, multi-systemic, clonal entity coalescing both neoplastic and inflammatory elements and strongly dependent on impaired *RAS/RAF/MEK/ERK* signaling.

## Introduction

Erdheim Chester disease (ECD) is a rare type of non Langerhans cell histiocytosis [1,2]. It is chiefly characterized by the migration and infiltration of lipid laden CD68(+), CD1a(-) histiocytes to various target organs resulting in the disruption of tissue architecture and fibrosis, thus causing impaired organ function and often bone pain [3]. Among different individuals, this condition may manifest in a heterogeneous spectrum of severity ranging from a mild to a life threatening disease [4]. Different patients may also present with an assortment of symptoms correlating with their specific sites of infiltration. However, up to 96% of patients exhibit radiological involvement of the skeleton [5]. While ^99m^Tc bone scintigraphy and positron emission tomography/computed tomography (PET/CT) are commonly used in the diagnosis of ECD, bone magnetic resonance imaging (MRI) may serve as a sensitive and valuable tool in the evaluation of cancellous bone involvement [6]. Apart from the skeletal system, other sites of involvement include the central nervous system [7-11], heart and great vessels [12-15], lungs [16-18], kidneys and retroperitoneum [19-23], adrenal glands [24], skin [25-27], gastrointestinal tract [28-31], breast [32-34], skeletal muscle [32,35], thyroid and testis [36]. Hemophagocytosis was also documented in the context of ECD [37]. Involvements of the central nervous system (CNS) and cardiovascular systems, in particular, were reported to be associated with an overall poor prognosis [38]. This is important to emphasize since the cardiovascular manifestations of ECD are frequently overlooked [39]. Although the mean age of diagnosis among ECD patients is 55 [5], pediatric cases have been documented [40-44]. To date, the etiology of ECD remains unknown and its pathogenesis, poorly understood. Much debate has arisen relating to the clonality of ECD [45-48]. On the one hand, it is well established that ECD is a phenomenon involving a dominant Th1 immune response, suggesting a dysregulated, inflammatory mechanism [49]. On the other hand, the *V600E BRAF* mutation recently identified in 54% of the patients [50] necessitates a precursor cell harboring this somatic mutation and, thus, a clonal neoplastic origin. A novel approach is described by Berres *et al*., who suggest that ECD is an example of an ‘inflammatory myeloid neoplasm’ [51]. The fitting therapeutic roadmap for the ECD patient is a complex mosaic of various clinical elements [52,53]. A diverse compilation of pharmacology has been under investigation over the past years in an attempt to strike at the Achilles heel of the disease. Currently, interferon-α is the sole agent which has demonstrated an increase in survival and is, thus, considered the first line of treatment [14,38,54-56]. Various second line treatments exist. Anakinra, an interleukin-1 receptor antagonist is suitable for mild disease [13,40,57-59]. Infliximab, an anti TNF-α monoclonal antibody has shown efficacy in the treatment of the cardiovascular involvement of ECD [60]. Vemurafenib, a small molecule which inhibits the *V600E* mutated *BRAF* protein, was found to induce remarkable responses among patients who harbor that mutation [61,62]. Finally, cladribine may be a reasonable therapeutic alternative for patients with moderate to severe disease who failed previous second line regimens [63-66]. Other treatments include various types of chemotherapeutic agents [67-70], radiation therapy [71-74], steroids [38,54,70,75], bisphosphonates [76-78] and bone marrow transplantation [79,80]. The prognosis of ECD is poor. Most patients suffer from progressive morbidity, which may relate to both the disease itself and its treatments. As for mortality, according to the largest series published, the one- and five-year survival rates of ECD patients are 96% and 68%, respectively [38].

### Patients and methods

Seven patients (five men, two women) were recruited to the study from six different medical centers in Israel (Table 1). Of these seven patients, five are discussed in detail. Each patient was evaluated and audited according to his/her disease distribution. Patients were included in this study subsequent to meeting the following inclusion criteria: first, a pathological confirmation was obtained only after the identification of CD68(+), CD1a(-) histiocytes in a biopsy specimen harvested from a locus of clinical interest. Second, a thorough, professionally performed workup of the patient was completed. This workup must have included a detailed anamnesis and a meticulous physical examination. Laboratory investigations must have included at least one complete blood count and one full blood chemistry panel. In regards to imaging, the patient must have undergone at least one CT scan of the thorax, abdomen and pelvis and one brain MRI. Cardiac MRI was performed only in patients suspicious of cardiovascular involvement on the basis of suggestive symptoms or prior imaging. As for nuclear medicine, at least one ^99m^Tc bone scintigraphy or one fluorodeoxyglucose (FDG) PET scan must have been performed. Molecular analyses of the *BRAF* oncogene were performed in six of the seven patients using digital PCR amplification followed by sequencing of the amplified segments using either Sanger’s sequencing technique or pyrosequensing, depending on the medical center in which each examination was performed.

**Table 1:** **Characteristics of the seven ECD patients**

**#**	**Age**	**Sex**	**Presenting sign/symptom**	**Disease involvement sites**	**BS**	**CT**	**PET/CT**	**Brain MRI**	**Heart MRI**	**Treatment roadmap**	**Notable responses and timeframe**	***BRAF*** **status**
1	39	F	Bone pain	STN, CNS, PIT, ROS, CUT, RP, PLM, RTN	+	+	+	+	-	STR → VB → IFNa → IFNa + VB → ANK → CLD → VMR	VMR: marked neurological improvement in 3w	+
2	50	M	Skin lesions and bone pain	STN, CNS, PIT, CUT, RP, ADR, LYM	+	+	-	+	-	CLD	CLD: regression of intracranial perivenous lesion and neurological stabilization, gradually over 13 m	-
3	62	F	Bone pain	STN, PIT, ROS, CUT, PLM, CV	+	+	+	+	+	STR → MTX + STR → IFNa + VB → VB → INX + MTX	INX + MTX - reduction in pericardial effusion volume over a 2 m period	-
4	55	M	Ataxia and dysarthria	STN, CNS, PIT, MNG, RP	-	+	+	+	-	IFNa	-	UNK
5	53	M	Ataxia and dysarthria	STN, CNS, PIT, MNG	+	+	+	+	-	STR → VB → pIFN → CLD	-	-
6	49	M	Abdominal pain	STN, PIT, ROS, RP, PLM, CV, GI	+	+	+	+	+	STR + VB + IFNa → VB + IFNa → IFNa → pIFNa → VMR → pIFNa	VMR: marked skin toxicity even at low doses	+
7	58	M	Polyuria, polydipsia and HTN	STN, PIT, CUT, RP, PLM, CV	+	+	+	+	+	IFNa	IFNa: Normalization of constitutional symptoms over a 6 m period	+

ADR, adrenal glands; ANK, anakinra; BS, ^99m^Tc bone scintigraphy; CLD, cladribine; CNS, central nervous system; CT, computed tomography; CUT, cutaneous involvement; CV, cardiovascular system; F, female; GI, gastrointestinal tract; HTN, hypertension; IFNa; interferon alpha; pIFNa, pegylated interferon alpha; INX, infliximab; LYM, lymph nodes; m, month; M, male; MNG, meninges; MRI, magnetic resonance imaging; MTX, methotrexate; PET/CT, positron emission tomography/computed tomography; PIT, pituitary gland; PLM, pulmonary system; ROS, retro orbital space; RP, retroperitoneum; RTN, retina; STN, skeleton; STR, steroids; UNK, unknown; VB, vinblastine; VMR, vemurafenib; w, weeks.

## Case reports

### Patient #1: a longstanding disease with multiple system involvement and a dramatic response to vemurafenib

**Figure 1:**
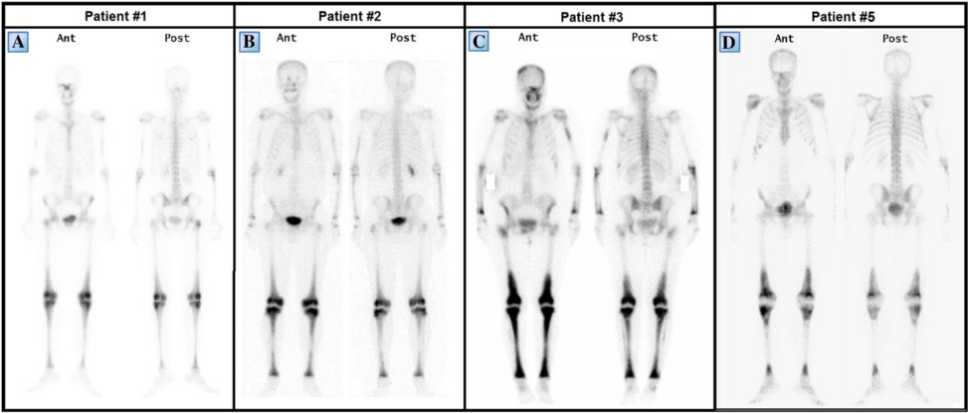
^**99m**^ **Tc bone scintigraphs of patients #1 (A), #2 (B), #3 (C) and #5 (D) taken prior to diagnosis.** Note the characteristic bilateral symmetric pattern of increased tracer uptake, particularly involving the femoral and tibial long bones and the periarticular regions of the knees. Despite an obvious variability in the degree of tracer uptake among patients in these series, the intensity of tracer uptake did not necessarily correlate with the degree of bone pain at the time of presentation.

**Figure 2:**
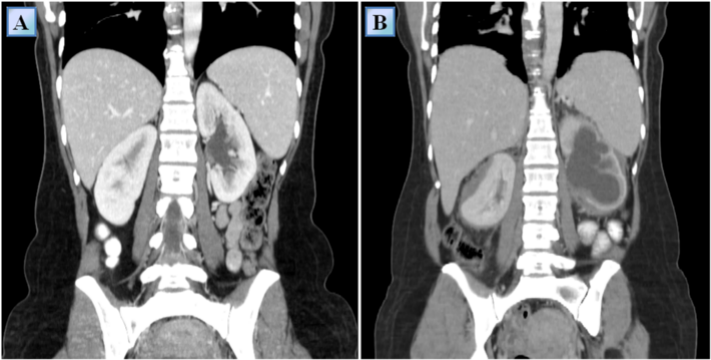
**Coronal reformatted contrast enhanced computed tomography images of patient #1, obtained at the time of the diagnosis (A) and 4.5 years after the diagnosis (B).** The latter reveals severe hydronephrosis and marked cortical thinning of the left kidney and a peri-renal mass compressing the right kidney. Also, note the fine bilateral peri-renal infiltrate forming a ‘hairy kidney’ appearance.

**Figure 3:**
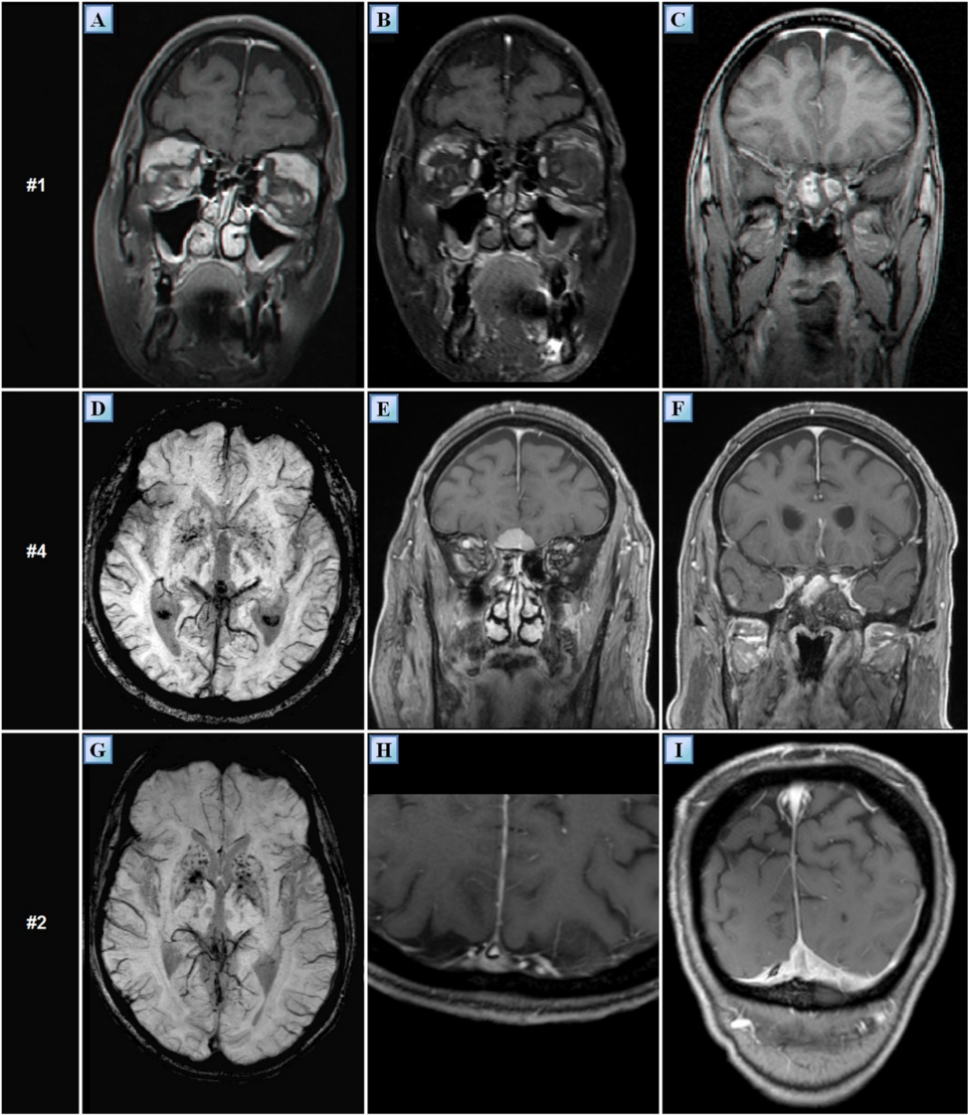
**Various intracranial MRI findings of patients #1, #2 and #4.** Coronal T1 weighted, gadolinium enhanced MR images of the retro-bulbar regions of patient #1 one year following diagnosis **(A)** and 4.5 years after diagnosis **(B)**. The former **(A)** demonstrates bilateral nodular masses located at the superior lateral aspect of the retro-orbit. These masses involve both the lacrimal glands and superior rectus muscles and undergo heterogeneous enhancement following gadolinium administration. The latter **(B)** demonstrates a marked reduction in the size of these lesions, presumably due to treatment with interferon-α. **(C)** Coronal T1 weighted, gadolinium enhanced MR image of patient #1 showing mucosal thickening in the sphenoidal sinus as well as soft tissue fullness which undergoes enhancement following gadolinium administration. This finding is also apparent in patient #4 **(F)**, who also exhibits an extra axial enhancing lesion situated in the vicinity of the planum sphenoidale **(E)**. In SWI sequence, both patients #4 **(D)** and #2 **(G)** exhibit multiple punctate hypointensities in the basal ganglia. These patterns are not typical for senile calcifications. Patient #2 also exhibits infiltrative enhancing tissue which narrows the transverse left sinus, adjacent to the falx and tentorium **(I)**. The same tissue is seen displacing the superior sagittal sinus **(H)**. MR, magnetic resonance; SWI, susceptibility weighted imaging.

**Figure 4:**
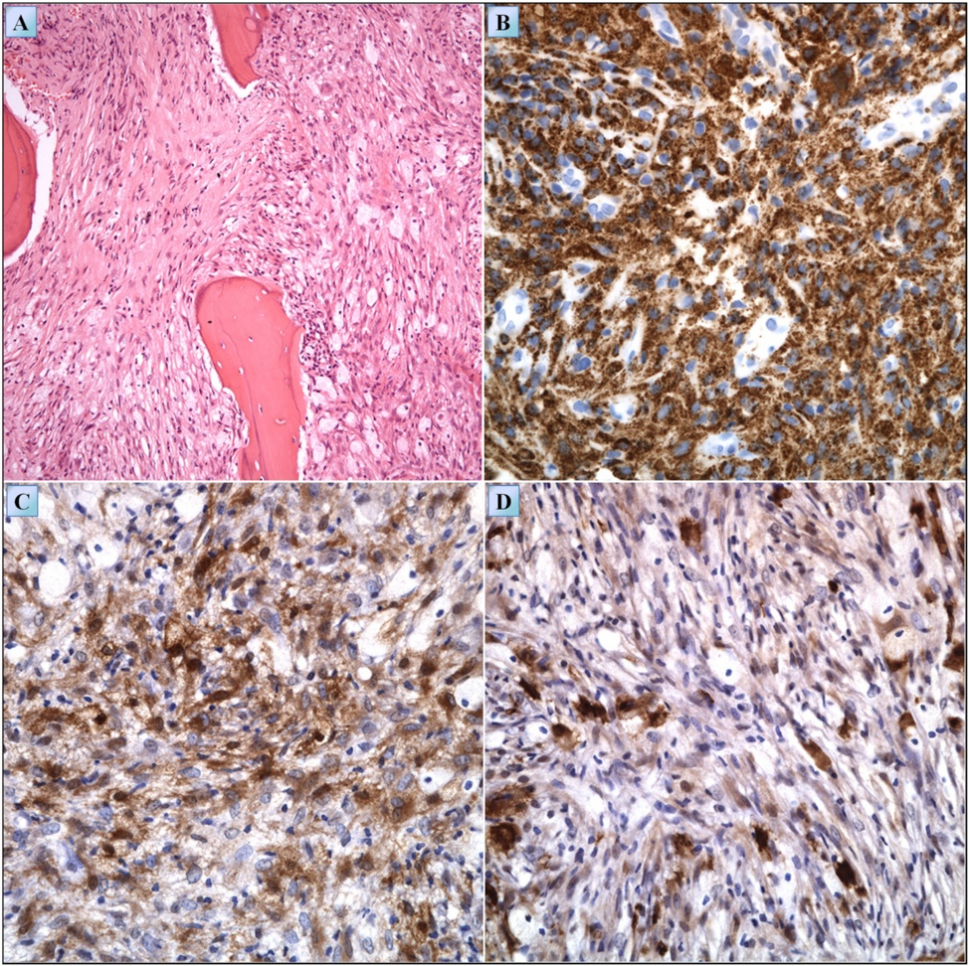
**Histological sample from the tibia of patient #1. (A)** H&E. Bony trabeculae separated by fibrosis and sheets of foamy macrophages. **(B)** Histiocytes demonstrating intense granular staining for CD68. **(C)** Histiocytes demonstrating patchy staining for factor XIIIa. **(D)** S-100 protein staining of the biopsy specimen.

A 39 year-old woman of Turkish-Iraqi descent presented in September 2007 to the emergency department with low grade fever, signs of weakness and difficulty walking due to diffuse bone pain mostly in the knees and both pectoral and pelvic girdles. Shortly before her admission the patient began experiencing alternating hot flashes and cold bouts disproportionate to the weather or clothing and suffered from frequent night sweats. Approximately six months prior to this incident, fatigue and leg pain slowly developed. The pain originally began as a tingling sensation, accompanied by paresthesia. It had escalated over several months and progressed to fully fledged bone pain. At the time of presentation, the pain was described as initiating at rest and constant, occasionally accompanied by episodes of fever. The patient was admitted for several consecutive hospitalizations. Upon inspection of her face, both exophthalmos and yellowish peri-orbital xanthelasmae-like lesions were apparent. The remainder of her physical examination was unremarkable, without deformation or bony swelling and without neurological deficits. It is noteworthy that the patient was polydypsic and polyuric for approximately five years prior to the onset of bone pain, consuming and excreting up to 12 liters of water per day. Despite this, a diagnosis of central diabetes insipidus was established and treatment with intranasal desmopressin initiated only upon hospitalization, presumably due to the patient’s adjustment to the gradual changes in water metabolism over the years. Apart from her known iron deficiency anemia, laboratory studies revealed increased levels of C-reactive protein (CRP: 70 mg/L) and elevated erythrocyte sedimentation rate (ESR: 72 mm/hour). A broad panel of immunologic studies, including rheumatoid factor (RF) and anti-nuclear antibodies was unremarkable. Negative blood and urine cultures ruled out an infectious etiology. Protein electrophoresis (PEP) yielded no monoclonal spike and the oncological markers were found to be within normal limits. Further investigations included radiography, which revealed bilateral sclerotic changes in the femoral and tibial bones and electromyography, which demonstrated normal muscle and nerve functionality. A subsequent ^99m^Tc bone scintigraphy demonstrated increased tracer uptake in the superior aspect of both orbits, the central sphenoidal region, left humeral head, right femoral greater trochanter and lateral superior aspect of the left iliac crest as well as diffuse, symmetric, bilateral increased tracer uptake in the femurs and tibiae enveloping both knees (Figure 1A). A CT of the femurs and tibiae exhibited an irregular osseous texture demonstrating bones riddled with mixed patchy lesions of sclerotic and lytic nature. This was suggestive of either a metabolic, malignant or granulomatous process. An additional CT of the thorax, abdomen and pelvis revealed congestion of both renal sinuses with slightly distended ureters (Figure 2A). CT and later MRI of the brain revealed bilateral retro-orbital nodular lesions (Figure 3A), diffuse thickening and tortuosity of the optic nerves, enlargement of both lacrimal glands and soft tissue fullness of the sphenoid sinus (Figure 3C). An MRI dedicated to the pituitary revealed no deviation from the normal anatomy. Over the two week period between two consecutive hospitalizations papilledema developed, accompanied by headaches, blurred vision and nausea. A lumbar puncture revealed an increased opening pressure of 44 cmH_2_O. Treatment with acetazolamide (250 mg X3 daily) was initiated, the increased intracranial pressure normalized and related symptoms abated. Biopsies were obtained from the tibial bone marrow and from a lacrimal gland. A microscopic examination of the tibial biopsy revealed bony trabeculae separated by a xanthomatous, diffuse spindle cell macrophage population with few interspersed giant cells (Figure 4A). The spindle cells stained relatively uniformly for the presence of CD163, and in a more patchy fashion but quite strongly for factor XIIIa (Figure 4C), surface staining for CD14 and granular intense staining for CD68 (Figure 4B). CD1a and S-100 staining were globally negative (Figure 4D) except for one focus of S-100(+) cells, suggesting infiltration of the marrow with Langerhans cells. Overall, these findings were most consistent with a diagnosis of ECD with a component of Langerhans cell histiocytosis. Over the five years pursuant to her diagnosis on May 2007, the patient was treated with several treatment protocols. At first, steroids (prednisone, 60 mg/day) provided a temporary relief - a marked decrease in bone pain, decrease in CRP levels and normalization of fever. Two months later vinblastine (6 mg/m^2^/week) was introduced as a steroid sparing agent. This protocol failed to evoke a favorable long lasting response and was substituted for treatment with interferon-α. Since then, interferon-α has served as the primary therapeutic agent administered at dosages as low as 3 × 10^6^ IU X 3 times weekly and as high as 6 × 10^6^ IU X 5 times weekly. Initially, interferon-α was administered as a single agent. Later on, it was complemented by vinblastine (8 mg/1 to 4 months) and by pamidronic acid (60 mg/1 to 3 months) in order to better control the lytic lesions. It is noteworthy that this patient continued to deteriorate neurologically even at the highest doses of interferon-α administered (6 × 10^6^ IU X 5 times weekly). However, the addition of vinblastine to this regimen succeeded in stabilizing the patient clinically and allowed for a gradual decrease in the dosage of interferon-α to a maintenance dose of 6 × 10^6^ IU X 2 times weekly over a two year period. Three years after the diagnosis of ECD, a four-month trial of anakinra (100 mg/day) was attempted, mainly due to multifocal disease progression. However, this agent yielded unsatisfactory results. This attempt was followed by a five-month trial of cladribine (0.14 mg/kg/day for five consecutive days, every four weeks), a drug which yielded no apparent benefit to the patient and was very poorly tolerated. By this time, the patient’s neurological symptoms, vis-à-vis her lingual and motor capabilities, had deteriorated greatly once again. She was markedly dysarthric and confined to a wheelchair. Consequently, a biopsy obtained from the peri-renal mass was attempted and found to contain ECD histiocytes which harbor the *V600E BRAF* mutation. After receiving proper counseling and signing an informed consent, treatment with vemurafenib (1,920 mg/day later tapered to 960 mg/day) was initiated with no less than a spectacular improvement. After two weeks of treatment the patient began to exhibit clinical improvement. Ultimately, following treatment with this agent, the patient regained her ability to walk distances of up to 500 meters and recovered her ability to speak fluently.

### Patient #1 - clinical analysis

Overall, the patient’s tolerance and response to treatment were the prime variables taken into consideration in the formulation of her therapeutic roadmap. Since the initial presentation of symptoms and throughout the different therapeutic lines, several significant radiological and clinical events had occurred.

**Figure 5:**
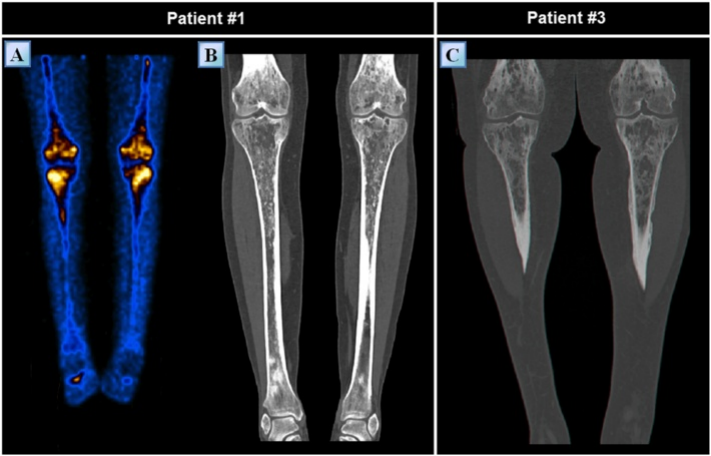
**PET and CT findings of the lower limbs of patients #1 and #3 (A)** Positron emission tomography showing symmetric, bilateral, abnormally increased intra-medullary uptake of fluorodeoxyglucose in the femurs and tibiae of patient #1 approximately 4.5 years following diagnosis. **(B)** Computed tomography of the femurs and tibiae of patient #1 at the time of diagnosis, exhibiting periostitis as well as diffuse, irregular intra-medullary sclerosis. These bones are riddled with mixed patchy lesions of sclerotic and lytic nature encased in a markedly thickened cortex. **(C)** Similar findings can be observed in the computed tomography of patient #3.

First and foremost, following treatment with interferon-α, the patient experienced complete resolution of the bone pain. Nevertheless, consecutive CT and PET/CT scans revealed deterioration of the osteosclerotic processes in the long bones as well as intense bilateral periarticular tracer uptake indicative of an active disease (Figure 5A,B). This was further substantiated by a gradual increase in bone density as shown on annual DXA (duel energy x-ray absorptiometry) assays aimed at assessing the bone density of the femurs, pelvis and spine.

**Figure 6:**
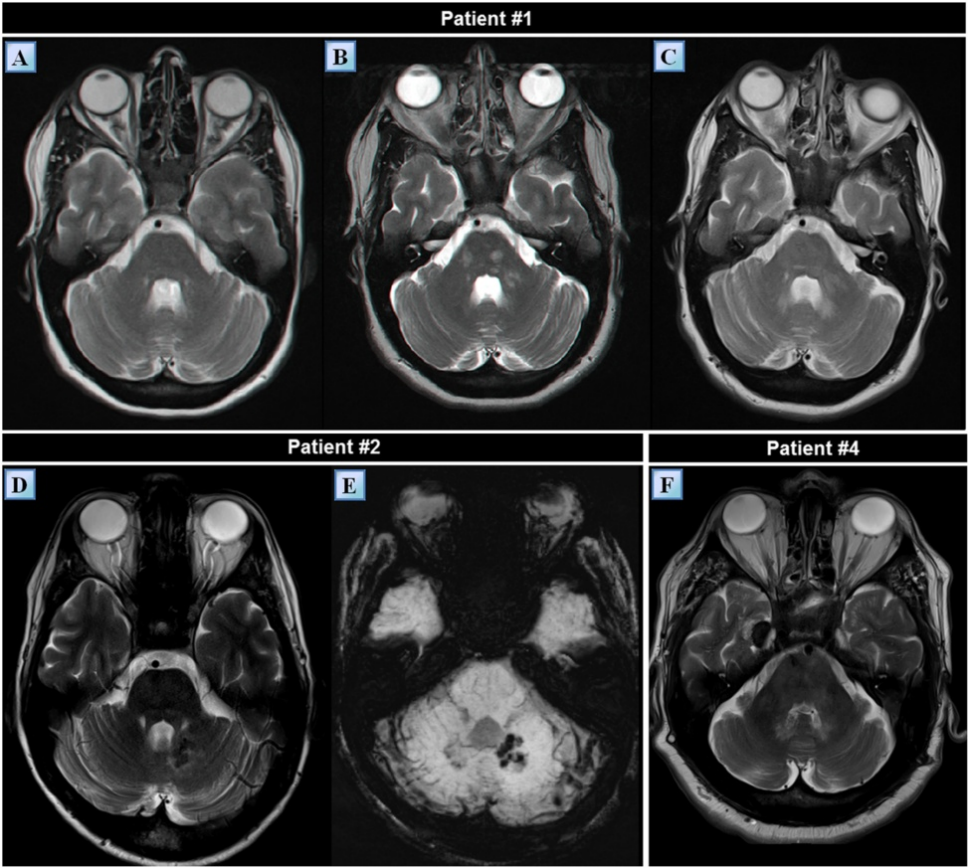
**Cerebellar MRI findings of patients #1, #2 and #4** Axial T2 weighted MR images of patient #1 at the level of the cerebellum, middle cerebellar peduncles and pons taken at the time of diagnosis **(A)** one year following diagnosis **(B)** and four years following diagnosis **(C).** Note the bilateral hyperintense signals which emerged one year following diagnosis in the pons and peri-dentate regions as well as in the left middle cerebellar peduncle **(B)**. The signal intensity of these processes seems to have decreased during the following three years **(C). (D)** An axial T2 weighted MR image of patient #2 at the level of the cerebellum demonstrating a unilateral focus of hypointense signal at the left peri-dentate region. **(E)** This cluster like lesion is better accentuated in SWI sequence. **(F)** An axial T2 weighted MR image of patient #4 displaying multiple, bilateral, diffuse foci of hyperintense signals involving the brainstem and peridentate areas. Another abnormal finding, adherent to the dura of Meckel’s cave, exhibits radiological similarity to a meningioma. Note the variable MR manifestations of cerebellar lesions in different ECD patients. ECD, Erdheim Chester disease; MR, magnetic resonance; SWI, susceptibility weighted imaging.

Second, one year following diagnosis, the retro-orbital lesions achieved maximal size (Figure 3A), with one lesion protruding extraconaly through the inferior orbital fissure causing inferior displacement of the orbit. The severe clinical progression necessitated the initiation of an aggressive combination treatment protocol comprising high dose interferon-α (9 × 10^6^ IU X 3 times weekly), vinblastine (8 mg/month) and pamidronic acid (60 mg/month). This treatment protocol resulted in marked regression of the retro-orbital lesions and was clinically evident by normalization of the exophthalmos and improvement of visual acuity (Figure 3B). The treatment also resulted in partial regression of the soft tissue fullness that originally developed inside the sphenoidal sinus. However, in parallel to the regression of the lesions mentioned above, new lesions have begun developing in concert with the interferon-α treatment. These new lesions appeared to be bilateral and to involve the cerebellar peri-dentate region, the middle cerebellar peduncles and the white matter of the pons (Figure 6A-C). Three years after her diagnosis, the patient’s ataxia and dysarthria progressed markedly.

**Figure 7:**
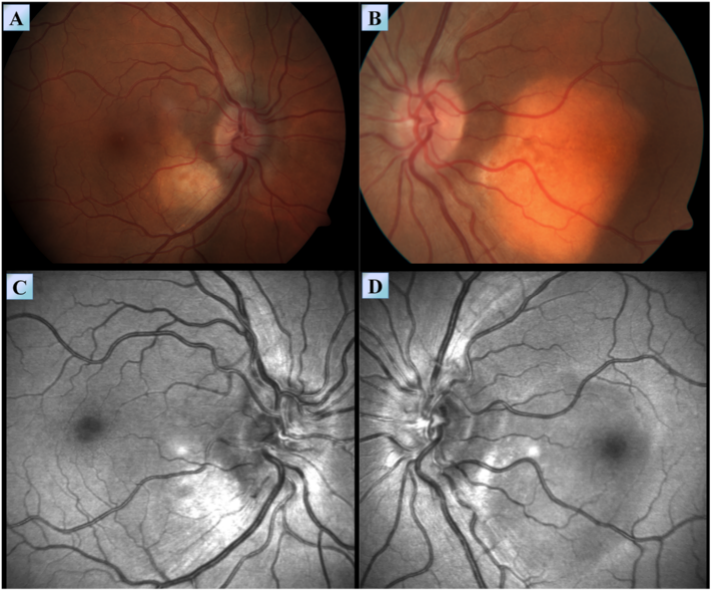
**Fundoscopy of patient #1.** Fundoscopy images of the right **(A)** and left **(B)** eyes of patient #1, demonstrating sub choroidal lesions involving the macula densa. **(C,D)** Red free imaging produced by a blue wavelength confocal scanning laser ophthalmoscope demonstrating the extent of these lesions in the right **(C)** and left **(D)** eyes.

Third, frequent fundoscopies were performed due to the patient’s tendency to develop papilledema in conjunction with episodes of increased intracranial pressure. Four years following diagnosis, new sub-choroidal lesions involving the macula densa appeared on fundoscopy of both eyes (Figure 7A-B). These findings were even more visible on red free imaging produced by a blue wavelength confocal scanning laser ophthalmoscope (Figure 7C-D). These findings proved to be stable on a neuro-ophthalmological follow up performed two years following their discovery. Thus, the initial visual field disturbances of patient #1 were attributed to increased intracranial pressure while those that appeared later, were attributed to these subchoroidal lesions.

Fourth, despite aggressive treatment, over the five-year period since diagnosis infiltration of the retroperitoneum had occurred with formation of a mild, bilateral ‘hairy kidney’ appearance on CT (Figure 2B). The left kidney underwent severe hydronephrosis with marked cortical thinning and the right kidney became compressed by a peri-renal mass. It is noteworthy that this mass was characterized by an increased tracer uptake on PET/CT. Globally, at the time of follow up, renal function is preserved with creatinine levels within normal limits.

Overall, a five-year follow up reveals favorable responses to treatment with interferon-α in terms of bone pain amelioration and regression of the retro-orbital lesions (Figure 3B). However, this agent had little effect on the retroperitoneal, central nervous system and ophthalmological involvements of the disease. Ultimately and most importantly, the patient exhibited dramatic improvement in neurological function following her treatment with vemurafenib.

### Patient #2: a cutaneous presentation with intracranial perivenous involvement responding to cladribine

**Figure 8:**
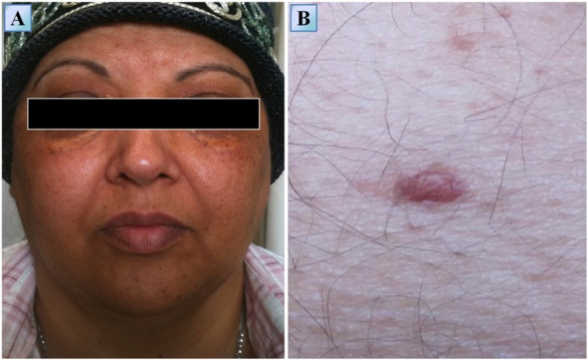
**Skin lesions of patients #2 and #3. (A)** Yellowish periorbital xanthelasma-like lesions in patient #3. **(B)** A red yellow papule, 8 mm in size, over the right flank of patient #2.

A 50-year-old man of Iraqi descent presented to an outpatient dermatology clinic in September 2010, complaining of asymptomatic skin lesions which had been recurring and remitting over the past seven years (Figure 8B). Upon an elaborated history, the patient disclosed suffering from mild lower limb pain, loss of libido and increased thirst and urination for the past seven years, as well. On examination, 10 red-yellow papules, 4 to 8 mm in size, were observed over his trunk and extremities. Laboratory findings revealed decreased levels of testosterone (3 nmol/L), lutenizing hormone (LH) (0.3 mIU/mL) and follicle-stimulating hormone (FSH) (0.4 mIU/mL). A 24 hour urine collection concluded in increased urine volume (3.2 L) and decreased urine osmolality. These findings led to the diagnosis of central diabetes insipidus and hypogonadotropic hypogonadism; thus, the patient initiated treatment with intranasal desmopressin and monthly intramuscular injections of choriogonadotropin alfa. Other laboratory tests including a complete blood count, thyroid hormones, lipid profile, viral serologies, protein electrophoresis, purified protein derivative (PPD), angiotensin-converting enzyme (ACE) and tumor markers were without pathological findings. Several imaging studies were conducted. Plain radiography demonstrated marked osteosclerosis of the tibiae and fibulae. A ^99m^Tc bone scintigraphy revealed intense symmetric bilateral uptake of tracer in the diametaphyseal regions of the distal femurs and proximal tibiae (Figure 1B). A brain MRI demonstrated thickening of the pituitary stalk and several cluster-like nodular enhancing lesions involving the left cerebellum in close proximity to the fourth ventricle (Figure 6D,E). Also, multiple punctate hypointensities were noted in the basal ganglia in SWI sequence (Figure 3G). This pattern is not typical of senile calcifications. The most prominent finding in this patient’s workup was that of intracranial soft tissue infiltrating posterior to the confluence of sinuses and sheathing the superior sagittal and left transverse sinus (Figure 3H-I). A whole body CT scan showed infiltration of the perirenal fat and bilateral enlargement of the adrenal glands. Three biopsies were eventually conducted: one of a skin lesion and two bone marrow biopsies. Surprisingly, both of the bone marrow biopsies exhibited normal bone marrow. However, the skin biopsy revealed histiocytes of varied morphology embedded in a lymphocytic infiltrate. The histiocytes stained positive for CD68 and negative for CD1a and Factor XIIIa. Electron microscopy of the specimen showed clusters of lipid droplets, with no evidence of Birbeck granules. These pathological findings were supportive of the diagnosis of ECD. Since his diagnosis in October 2010, the patient deteriorated on several occasions, exhibiting a repertoire of signs and symptoms. Among them were both constitutional and neurological symptoms. The various emerging phenomena included episodes of increased intracranial pressure with papilledema, ataxia, dysarthria, pathological crying and laughing, hyper-reflexia and nystagmus. The variety of these symptoms prompted a more thorough evaluation of cerebral blood flow. Both CT venography and later MR venography demonstrated attenuated flow in the superior sagittal sinus due to external compression on the vessel, displacing it from the skull. Also, a complete collapse of the left transverse sinus was noted. Over the three years following the diagnosis the patient was treated with monthly four to five day courses of cladribine IV, administered intravenously at dosages of 4.5 to 4.8 mg daily (0.07 mg per kg of body weight daily). The patient received a total of 13 courses of cladribine, which resulted in a significant improvement in his fatigue and weakness. In addition, a decrease in the episodes of papilledema and stabilization of his neurological symptoms were noted along with an evident radiological decrease in the amount of infiltrative tissue compressing the superior sagittal sinus.

### Patient #2 - clinical analysis

Several key issues should be addressed in the analysis of this patient. First, this patient exemplifies how elusive the diagnosis of ECD can be. He originally sought medical attention in an outpatient clinic to investigate his recurring dermatological lesions. A thorough workup which originated in a detailed history performed by his physicians succeeded to lift the veil off the relatively subtle symptoms that were evident in the initial phases of his disease and to associate them with his skin lesions.

Second, in this particular case, an extra-axial, perivascular intracranial process appears to impair the cerebral blood flow, causing both chronic indolent damage as well as acute flare-ups of increased intracranial pressure. It may very well be that this patient’s neurological symptoms, originate both from global oxygen deprivation as well as focal disease-associated lesions. This is evident due to the nature of this patient’s symptoms, as he exhibits signs of upper motor neuron impairment and episodes of increased intracranial pressure alongside pseudobulbar and cerebellar syndromes. Additionally, treatment induced lesion regression seems to slightly alleviate and stabilize the global neurological impairment while the focal deficits remain unchanged.

Third, one must consider that several biopsies may be needed to harvest a satisfactory specimen. Thus, a negative biopsy should not exclude a working diagnosis of ECD in otherwise highly suggestive settings.

Finally, this patient received prolonged treatment with lower doses of cladribine in an attempt to control his CNS involvement. Although considered an advanced line treatment in ECD, this treatment strategy appears to have resolved this patient’s constitutional symptoms and caused regression of the lesions sheathing his intracranial vasculature.

### Patient #3: a case of severe cardiovascular involvement stabilized by treatment with infliximab

A 55-year-old woman of Algerian descent presented to our medical center in August 2008 with a chief complaint of worsening bone pain. She began suffering from a mild degree of diffuse bone pain 13 years ago. The pain was described as constant and as affecting her lumbar spine, knees, pelvic bones and feet. The pain was managed rather well until her time of presentation using non-steroidal anti-inflammatory drugs. At the time of presentation, the pain had intensified to a degree which prevented her to stand from a sitting position. The patient had a past medical history of diabetes insipidus for which she was diagnosed 12 years prior to her current presentation and also suffered from recurrent urinary tract infections. Her physical examination was unremarkable aside from periorbital xanthelasma-like lesions (Figure 8A). Abnormal laboratory findings included a microcytic iron deficiency anemia (hemoglobin: 11 g/dL, MCV: 77.5 fL, iron: 19 μg/dL) and an elevated ESR (98 mm/hr). Both protein electrophoresis and tumor markers were without pathological findings. A radiograph of the lower limbs was obtained and demonstrated bilateral involvement of the femurs and tibiae with cortical and periosteal thickening accompanied by a mixture of lytic and sclerotic lesions. These findings were even more apparent on CT (Figure 5C). A ^99m^Tc bone scintigraphy revealed a multifocal bone disease characterized by bilateral, symmetric increased tracer uptake involving both tibiae, distal femurs and iliac bones. Also, an asymmetric increased tracer uptake was noted in both of the upper limbs, favoring the right humerus, radius and ulna (Figure 1C). A CT guided biopsy of the right femur was performed. The specimen included cores of long bone showing focal fibrosis, foamy histiocytic infiltrate, inflammatory cells, giant cells and reactive cortical sclerosis. The histiocytes described were found to be CD68 positive and CD1a negative, a histological picture compatible with ECD. After her diagnosis was established in December 2009, the patient was treated with prednisone at an initial dosage of 60 mg/day which underwent gradual tapering to 5 mg/d over a period of one year. Five months after the initiation of prednisone, methotrexate was introduced at escalating dosages of 10 to 25 mg/week. This treatment protocol was successful in generating a transient decrease in bone pain which lasted for two years. However, during that period of time, the patient began experiencing extraskeletal symptoms: alternating hot flashes and cold bouts, blurred vision, headaches, nausea and vomiting. Last but not least, the patient’s most prominent complaint was dyspnea, as she suffered from frequent episodes of shortness of breath following even the slightest of physical efforts, rendering her unable to walk more than 100 meters. Assessment of the patient’s CNS included a brain MRI which was without pathological findings. The patient’s cardio-pulmonary evaluation included several echocardiography studies which revealed recurrent pericardial effusions of moderate to large proportions with associated fibrin. Contrast enhanced CT of the chest demonstrated periaortic infiltration (‘coated aorta’) of the aortic root as well as infiltration of the mediastinum adjacent to the right atrium and also confirmed the existence of a small loculated pleural effusion. In addition to the known pericardial effusion, gadolinium enhanced cardiac gated MRI revealed thickening of the pericardium, thickening of the wall of the ascending aorta (of up to 10 mm) and several lesions encasing the right atrium, right coronary artery and superior vena cava. These findings were also apparent on a whole body PET/CT scan which demonstrated increased tracer uptake in the lesions encasing the great vessels as well as in the osseous foci consistent with the former ^99m^Tc bone scintigraphy. Due to the multifocal progression of her disease, the patient was started on a combination therapy regimen of interferon-α (3 × 10^6^ IU X2 to 3/week) and vinblastine (6 mg/m^2^/week). However, this regimen was tolerated very poorly by the patient, as she became severely neutropenic with an absolute neutrophil count of less than 500 cells/μL. The dual interferon-α - vinblastine regimen was consequently stopped after four weeks of treatment and replaced with vinblastine as a single agent regimen at a dosage of 6 to 8 mg/m^2^/week. Nevertheless, even this treatment was poorly tolerated and, thus, was stopped following an additional eight weeks of therapy. In parallel, the patient was tested for the *V600E BRAF* mutation and was found to be negative. Ultimately, a combination therapy regimen of infliximab (5 mg/kg/6 weeks) and methotrexate (20 mg/week) was initiated. Following six courses of treatment the patient improved clinically, with a notable decrease in the frequency and intensity of her pain, nausea and dyspnea. A marked reduction in the proportion of the pericardial effusion was noted on both echocardiography and computed tomography. However, despite treatment, the mediastinal lesions were found to be metabolically active on PET/CT and no reduction in their size was evident on CT. Most interestingly, this treatment resulted in better control of the patient’s diabetes insipidus, necessitating a decrease in the dosage of desmopressin.

### Patient #3 - clinical analysis

This patient, who suffers from ECD with a prominent cardiovascular component, presents several medically noteworthy insights. First, recurrent, moderate to large pericardial effusions comprise a worrisome medical phenomenon. These effusions raise questions regarding the potential future compromise of cardiac function in the face of rapid effusion volume fluctuations. Therefore, we recommend that ECD patients with substantial pericardial effusions should be monitored frequently using echocardiography and assessed for potential pericardiocentesis should significant cardiac compromise occur. The architecture of the mediastinal lesions, their size and location, are important variables when considering invasive interventions. In patients with extensive cardiovascular involvement, cardiac MRI may serve as the modality of choice in monitoring their complex mediastinal anatomy [12]. Overall, ECD associated encasement of the cardiac and/or mediastinal blood vessels should be monitored with caution for potential signs of stenosis. For example, in this patient, the superior vena cava and right coronary artery are involved and, thus, a high index of suspicion should remain with respect to potential emergencies involving these vessels. Compromised flow in the superior vena cava (*vis-à-vis* the superior vena cava syndrome) could cause both cerebral and vocal cord edema, manifesting as hoarseness and facial edema alongside superficial venous distention on the neck and upper trunk. In this patient, treatment with infliximab succeeded in greatly reducing the volume of the effusion and, thus, improved the patient’s clinical status. As for her prior treatment regimen, we concluded that low dose methotrexate may be a reasonable treatment alternative in selected cases of mild disease free of extraskeletal manifestations, as this patient benefited from it for two pain controlled years. Conversely, steroids are no longer recommended as a first line of treatment for ECD.

### Patients #4 and 5: two cases of an atypical, painless presentation of ECD with intracranial involvement refractory to interferon-α

#### Patient #4

A 62-year-old man of Yemenite descent presented with a myriad of symptoms which underwent gradual worsening over the year prior to his evaluation in our medical facility in February 2012. Among his complaints were marked fatigue and weakness, difficulty speaking, disturbance in balance and depression. His past medical history included hypertension and type II diabetes mellitus. In addition, he was diagnosed ten years prior to our evaluation with diabetes insipidus, for which he was treated with nasal desmopressin. In the process of diagnosing diabetes insipidus, the patient underwent a brain MRI which revealed multiple meningioma-like lesions as well as a finding suspected to be a demyelinating pontine lesion. Since no neurological deficits were identified, the patient remained under surveillance until one year prior to our evaluation, when he began suffering from insidious pseudo-bulbar and cerebellar syndromes. His neurological examination revealed ataxia, dysarthria, dysmetria and dysdiadochokinesia. Laboratory findings included a microcytic anemia, with a mean corpuscular volume (MCV) of 64.8 fL. A brain MRI revealed three meningiomas, an olfactory meningioma, a frontal meningioma adjacent to the planum sphenoidale (Figure 3E) and a meningioma of the dura encasing the posterior aspect of the cavernous sinus, in the vicinity of Meckel’s cave. The scan also showed unenhanced, heterogeneous infiltration of the pons, upper medulla and dentate nuclei (Figure 6F) as well as thickening of the infundibulum accompanied by the absence of signal from the posterior pituitary. In addition, similar to patient #2, this patient exhibited a pattern of multiple punctate hypointensities involving the basal ganglia in SWI sequence (Figure 3D). These accumulated findings raised the possibility of a histiocytic disorder and, thus, prompted a PET/CT scan. The whole body PET/CT scan showed increased symmetric bilateral tracer uptake in the bones of the lower limbs. These bones demonstrated a patchy pattern of mixed lytic and sclerotic lesions. On CT, an abnormal appearance of the kidneys was noted and interpreted as infiltration of soft tissue into the region of the renal pelvises. Two biopsies were obtained from the tibial bones. The first bone marrow biopsy demonstrated a normocellular trilineage marrow with no evidence of histiocytosis. Only the second biopsy revealed fibrotic bone marrow lacking hematopoietic cells with clusters of CD68(+), foam cells as well as a mild to moderate mononuclear infiltrate. Several multi-nucleated giant cells were noted. Stains for CD1a and S-100 were negative. These findings were consistent with ECD. Following his diagnosis in May 2012, the patient began treatment with interferon-α at dosages of 1 to 3 million IU three times weekly. Despite the low dosages, this treatment was poorly tolerated. Over the 15 months period since his diagnosis was established, the patient’s disease progressed indolently. Shortly after, his neurological manifestations began deteriorating rapidly. In parallel, his performance status declined markedly, he became bedridden and succumbed to his disease three months after, an overall 18 months following his diagnosis.

#### Patient #5

A 53-year-old man of Libyan descent sought medical attention in November 2012 due to deterioration in his walking stability as well as slowing of his speech. Apparently, these symptoms slowly progressed over a period of approximately 18 months prior to this patient’s presentation. Upon a detailed history, the patient disclosed suffering from polyuria and polydipsia for the last 7.5 years. A prior medical investigation aimed at identifying the cause of these symptoms was concluded as non diagnostic. His neurological examination revealed dysarthria, dysmetria, gait instability and a multidirectional gaze evoked nystagmus. Also, decreased left flank muscle tone and lower limb hyporeflexia were noted. A fundus examination was unremarkable. Aside from a mild normocytic anemia (hemoglobin: 12.4 mg/dL, MCV: 86.6 fL), other laboratory studies, including a lumbar puncture, were without significant findings. Laboratory studies aimed at identifying viral serologies and paraneoplastic antibodies were negative. The conjunction of this patient’s cerebellar syndrome, polyuria and polydipsia raised a suspicion of a histiocytic disorder. A ^99m^Tc bone scintigraphy study was conducted and revealed bilateral, abnormally increased tracer uptake in the distal femurs and both proximal and distal tibiae (Figure 1D). A brain MRI uncovered several abnormal findings, including a pontine lesion, mild cerebellar atrophy, absence of the posterior pituitary and a parietal parasagittal extra-axial lesion 23 mm in diameter, consistent with a meningioma. A whole body PET/CT scan was conducted and disclosed an increased tracer uptake in the pons, as well as the femurs and tibiae. The PET findings in the long bones correlated well with osteosclerosis as seen on CT and with the areas accentuated on the previous ^99m^Tc bone scintigraphy study. A biopsy from the tibial bone marrow was performed. Microscopic examination of the specimen revealed thickened, irregular bony trabeculae with numerous cement lines, insinuating increased cellular turnover. The marrow itself was found to be diffusely fibrotic with a small amount of residual fat tissue and numerous macrophages which carry a CD68(+), CD1a(-), S-100(-) immunohistochemical profile. Langerhans cells and hematopoietic cells were not observed. This picture was consistent with bone marrow involvement of ECD. Following diagnosis in April 2013, the patient was tested for the *V600E BRAF* mutation and was found to be negative. Steroids were introduced as a first line treatment and later combined with vinblastine (6 mg/m^2^/week) as a steroid sparing agent. This treatment was administered for five months and subsequently ceased due to progressive neurological deterioration. Second line treatment with the pegylated form of interferon-α (135 μg/week) was administered for two months yet failed to halt this patient’s neurological deterioration. Ultimately, the patient initiated treatment with cladribine IV (0.14 mg/kg/day for five consecutive days, every four weeks). Following four cycles of treatment a mild degree of radiological improvement was noted on brain MRI. Nevertheless, no clinical improvement was noted, neurological or otherwise.

#### Patients #4 and 5 - clinical analyses

In these patients, aside from their longstanding diabetes insipidus, a well-known preceding sign of ECD, the initial investigations eliciting symptoms were purely neurological, primarily cerebellar in nature, without any involvement of bone pain. In both cases, a working diagnosis of a possible histiocytic disorder was raised due to a high index of suspicion, based on the clinical presentation, longstanding diabetes insipidus and on the findings of the brain MRI studies. These two patients exhibited a rather interesting phenotype of ECD, restricted primarily to the skeleton and central nervous system. Intracranially, both suffered from meningiomas and involvement of the ponto-cerebellar region. Nevertheless, a radiological subclinical focus of disease was apparent only in the retroperitoneum of patient #4. In terms of response to treatment, these patients exhibited a comparable response pattern as well as a suboptimal outcome. It is likely that higher dose interferon-α regimens may be necessary in ECD patients with severe CNS involvement, as previously documented by Hervier *et al*. [56].

## Results

Seven patients (five men and two women) with biopsy proven ECD were recruited from six medical centers in Israel. The median age at the time of presentation was 53 years (range: 39 to 62 years). The median follow-up time was 36 months (range: 1 to 72 months). Six of the seven patients (86%) suffered from diabetes insipidus that preceded the onset of investigation eliciting symptoms by a median time of 87 months (range: 9 to 144 months).

The different presenting symptoms included bone pain in 43% (three of seven) of the patients. This pain ranged from mild (one patient) to severe (two patients). The patient who presented with mild bone pain originally sought medical attention for relapsing remitting skin lesions in outpatient settings. An atypical, painless presentation characterized 43% (three of seven) of the patients, who presented with worsening neurological symptoms primarily of cerebellar nature (two patients) or with renovascular hypertension (one patient). One patient (14%) presented in the setting of an acute abdomen due to perforation of his appendix by histiocytic infiltrate. These different symptoms were accompanied by constitutional symptoms, such as fever, malaise and fatigue in all of the patients. Episodes of increased intracranial pressure notable by papilledema were evident in 57% (four of seven) of the patients. Preliminary complete blood counts identified a microcytic anemia in 57% (four of seven) of the patients, elevated levels of CRP in 43% (three of seven) and increased erythrocyte sedimentation rate in 28.5% (two of seven).

Bone involvement was radiologically obvious in all of the patients although bone pain ultimately affected only 57% (four of seven) of the patients. The bone lesions were mostly of mixed lytic-sclerotic nature. In addition, 57% (four of seven) of the patients suffered from CNS involvements of the disease associated with emerging neurological symptoms. These intracranial involvements included mostly lesions of the pons and cerebellum, but also lesions of the cerebral hemispheres, venous sinuses and meninges. The various clinical manifestations attributed to these lesions were ataxia, dysarthria, dysmetria, dysdiadochokinesia and disturbance of balance. Retro-orbital lesions caused exophthalmos in 43% (three of seven) of the patients. A ‘hairy kidney’ appearance on CT was identified in 71% (five of seven) of the patients. However, renal function was preserved in all of the patients. Patient #1 exhibited the worst renal involvement, with severe hydronephrosis and marked thinning of the cortex of the left kidney as well as compression of the right kidney by an FDG uptaking mass on a PET/CT scan. Skin lesions were noted in 57% (four of seven) of the patients. These were periorbital yellowish xanthelasma like lesions (three patients) and up to 8 mm in diameter nodules of yellow-red color (one patient). Pulmonary involvement was evident in 57% (four of seven) of the patients. Two suffered from pleural effusions and the other two exhibited mild thickening of the interlobular septae on CT. The mediastinum, heart and great vessels were involved in 43% (three of seven) of the patients. One example of cardiovascular involvement was found in patient #3 who exhibited encasement of the mediastinum and great vessels, lesions of the right atrium and recurrent pericardial effusions which responded to treatment with infliximab. Novel ophthalmological findings were identified in patient #1, who exhibited an infiltration pattern of the sub-choroidal layer on fundoscopy.

In order to confirm the diagnosis, an average count of 1.7 separate biopsies was required (range: one to three biopsy sites), with a positive biopsy obtained from a long bone source in 57% (four of seven) of the patients. Fifty percent of the patients tested for the *V600E BRAF* mutation (three of six) were found to harbor this particular mutation. The different therapeutic agents used were steroids, interferon-α, cladribine, vinblastine, methotrexate, anakinra, infliximab and vemurafenib. Benefit from treatment was considered as either normalization of the constitutional symptoms, clinical improvement correlating with the relevant sites of involvement, radiological evidence of lesion regression or a combination of these three parameters. Interferon-α was beneficial in 50% (three of six) of the patients treated. It is noteworthy that the peri-renal, cardiovascular and intracranial involvements proved to be rather refractory to treatment with interferon-α at dosages below 6 × 10^6^ IU X 3 times weekly. Additionally, notable neurological improvement was observed following treatment with vemurafenib in a *BRAF V600E* positive patient. Regression of a substantial pericardial effusion was observed following treatment with infliximab and moderate reduction in the compression of intracranial perivascular sheathing (evident by improved perfusion and imaging) was achieved after treatment with cladribine.

## Discussion

In the past three years, the constantly growing clinical awareness of medical professionals has globally promoted several substantial achievements in the evolution of ECD. First, the rate at which patients are being diagnosed has risen dramatically. Patients are being diagnosed much earlier in the course of their disease. We now witness subclinical, nearly asymptomatic ECD patients. Thus, new dilemmas are discussed regarding the potential benefits and risks of early medical interventions. Second, annual ECD conferences are being held, promoting novel research initiatives as well as the emergence of the first edition of consensus guidelines [53].

Nevertheless, the proper diagnosis and management of an ECD patient remains a challenge. From our clinical experience with seven patients, several key issues should be addressed in the management of such patients. In terms of diagnosis, as previously reported [81,38], diabetes insipidus of an unexplained etiology may precede the onset of investigation eliciting symptoms by as many as twelve years. Most peculiarly, the various anatomical disruptions of the hypothalamic - pituitary regions that could explain such a phenomenon appear on MRI, if at all, months to years following diagnosis. This gap of time suggests that the initial manifestations of ECD could indolently progress from microscopic involvement which generates a functional disturbance of the posterior pituitary. Ultimately, this process may become evident on MRI as absence of the high signal intensity of the posterior pituitary on T1 weighted images or as thickening of the pituitary stalk.

Another core issue relating to diagnosis of ECD is the investigation eliciting symptom, which is most commonly bone pain. Complaints of diffuse long bone pain of unexplained etiology in conjunction with a history of diabetes insipidus should prompt a high index of suspicion towards ECD. This suspicion may be further substantiated in the face of constitutional inflammatory signs and symptoms, such as fever, malaise and increased levels of ESR and CRP. The typical, symmetrical, bilateral appearance of increased tracer uptake involving the long bones on a ^99m^Tc bone scintigraphy study should prompt biopsies from high suspicion bone marrow foci for CD68(+), CD1a(-) histiocytes, a finding which finalizes the diagnosis of ECD. Additionally, a PET/CT scan may be useful in documenting extraskeletal involvements of ECD for the purposes of follow-up or tissue acquisition. Occasionally, more than one biopsy may be needed to successfully obtain a diagnostic specimen from a focus of interest. In many cases, the bone marrow comprises a reasonable biopsy site. This is because it has proven reliable in the detection of the typical tissue architecture that characterizes ECD on pathological examinations, as well as the relative ease at which immunohistochemical dyes are applied. However, detection of the *V600E BRAF* mutation using PCR technology is more difficult to perform and yields less accurate data when processed from a skeletal tissue source. Conversely, favorable specimen sources for such an examination are ECD skin lesions and the infiltrated peri-renal fat that appears as a ‘hairy kidney’ on computed tomography.

Renal involvement is a common finding in ECD. In their paper published in 2011, Haroche *et al*. report from their clinical experience that 68% of their patients were found to express a ‘hairy kidney’ appearance on imaging studies [5]. This radiological finding is typical of peri-renal histiocytic infiltration among ECD patients. Renal involvement in ECD should be a concern for the managing physician, as peri-renal lesions may ultimately lead to a post renal obstruction causing silent loss of a single, or an apparent loss of both, kidneys [21]. Moreover, peri-renal involvement in ECD may compromise the patency of the renal arteries due to external compression, causing secondary renovascular hypertension and renal failure [82]. Thus, frequent sonographic assessments of the kidneys and ureters are advised for ECD patients alongside ureteral stenting in selected cases.

The intracranial manifestations of ECD are diverse. However, involvement of the cerebellum is frequent, particularly in the peri-dentate regions and in close proximity to the fourth ventricle. Radiologically, these lesions may be heterogeneous in nature. It is noteworthy that two of our seven patients exhibited multiple punctate hypointensities in the basal ganglia in SWI sequence. This pattern was never observed by us before in the context of ECD. Yet another novel finding was that of an ophthalmological involvement of ECD. In patient #1, sub-choroidal lesions involving the macula densa appeared on fundoscopy of both eyes and were better visible on red free imaging produced by a blue wavelength confocal scanning laser ophthalmoscope. These findings proved to be stable on a neuro-ophthalmological follow up performed two years following their discovery. To our knowledge, this is the first report of ECD-associated retinal findings ever reported in the medical literature. This subchoroidal infiltration pattern may contribute to the understanding of the mechanisms involved in visual disturbances in ECD. Disturbances of vision in ECD are attributed to exophthalmos, increased intracranial pressure and papilledema. It is plausible that interruption of the visual fields and decreased visual acuity may originate in part due to a microscopic process involving the retina. In the case of patient #1, the initial visual field disturbances were attributed to increased intracranial pressure while those that appeared later, were attributed to these subchoroidal lesions.

Treatment wise, it is well established that interferon-α should serve as the mainstay of therapy for ECD. In the past, vinblastine was administered to ECD patients as a single agent or in combination with interferon-α with anecdotal reports of objective response [68,83]. Vinblastine was considered primarily by analogy to the treatment of Langerhans cell histiocytosis, potentially as a means to augment the effect of interferon-α. However, of three patients (#1,#3,#5) treated with vinblastine, only patient #1 benefited from the addition of vinblastine to high dose interferon-α.

Recent advances in the understanding of the molecular biology underlying ECD have led to novel therapeutic approaches to this disease. The accumulated data in the literature strongly advocate that ECD is, in fact, a clonal, neoplastic disorder originating from monocytic myeloid blood components that exhibit prominent inflammatory characteristics. A majority of these clones seem to be predominantly driven by molecular elements involving the *RAS/RAF/MEK/ERK* signal transduction pathway, such as *BRAF* and *NRAS*, among others [50,84]. One particular element identified in several large series, as well as in ours, is the occurrence of the *V600E BRAF* mutation in approximately 50% of the patients [50]. Nevertheless, the ability to accurately detect such genetic alterations is subject to several limiting factors including adequacy of samples, accuracy of the technology used and the level of staff expertise in performing such tests. For example, Cangi *et al*. report they were able to detect the *V600E BRAF* mutation in all of the patients evaluated in their cohort using the appropriate molecular techniques [85].

The pharmacological targeting of the *V600E BRAF* mutation by the drug vemurafenib repeatedly yielded dramatic clinical benefit in a significant portion of patients, unparallel to that of any other agent attempted in the treatment of ECD. Evidently, this mutation should be considered as a cardinal determinant in the therapeutic roadmap planning for ECD patients. From our limited experience, we support vemurafenib as a second line treatment agent for ECD patients who tested positive for the *V600E BRAF* mutation and failed to respond to adequate doses of interferon-α.

*BRAF* negative patients should be offered the repertoire of treatment alternatives according to the severity and distribution of their disease. In our series, interferon-α was found to play a beneficial role in the treatment of the skeletal manifestations of ECD. However, different patients in our series responded variably to interferon-α in terms of the extraskeletal disease involvement sites and the degree of lesion and symptom regression. A delicate balance between toxicity and efficacy should be sought in the dosage modulation of interferon-α, especially in patients with cardiovascular and CNS disease localizations.

### Patient consent

All the patients included in this study signed a consent form permitting the use of their medical records for the purpose of this publication.
